# Transparent Wood for Passive Radiative Cooling of Solar Absorbers

**DOI:** 10.1021/acs.nanolett.5c02994

**Published:** 2025-09-11

**Authors:** Farsa Ram, Martin Höglund, Mingna Liao, Tomas Hallberg, Magnus P. Jonsson, Lars A. Berglund, Ravi Shanker

**Affiliations:** † Wallenberg Wood Science Center, Department of Fiber and Polymer Technology, 7655 KTH Royal Institute of Technology, Teknikringen 56, 100 44 Stockholm, Sweden; ‡ Interdisciplinary Centre for Energy Research, 29120 Indian Institute of Science, Bengaluru-560012, India; § Laboratory of Organic Electronics, Department of Science and Technology, Linköping University, SE-601 74 Norrköping, Sweden; ∥ Department of Electromagnetic Signatures, 7644 FOI-Swedish Defense Research Agency, 583 30 Linköping, Sweden

**Keywords:** passive radiative cooling, thermal radiation, thiol−ene, transparent wood, atmospheric window, cellulose, zinc oxide

## Abstract

Passive radiative cooling is emerging as a sustainable strategy to reduce energy consumption by emitting heat directly through Earth’s atmospheric transparency window. Here, we demonstrate transparent wood-based biocomposite coatings as an eco-friendly solution for passive radiative cooling under direct sunlight. We fabricated freestanding, micron-thick coatings using wood scaffolds functionalized with ZnO nanoparticles, followed by thiol–ene in situ polymerization to improve transparency and mechanical resilience. These coatings exhibit high visible transparency combined with exceptionally strong mid-infrared emissivity (∼0.95). When applied onto silicon substrates exposed to direct sunlight, ZnO-functionalized coatings effectively lowered the substrate temperature by ∼6–7 °C. This was primarily attributed to enhanced thermal radiation, highlighting their potential for mitigating overheating in solar cells and other sunlight-exposed structures. Additionally, the enhanced mechanical properties of these biocomposites provide versatility for structural and optical applications, positioning them as a cost-effective, bio-based alternative to traditional cooling technologies.

## Graphical abstract

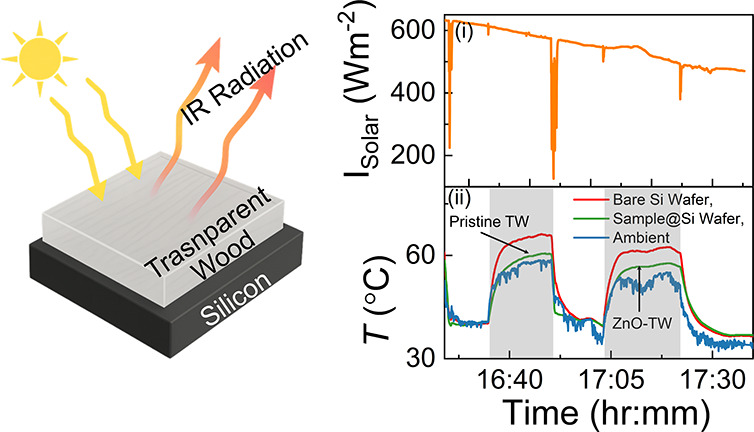

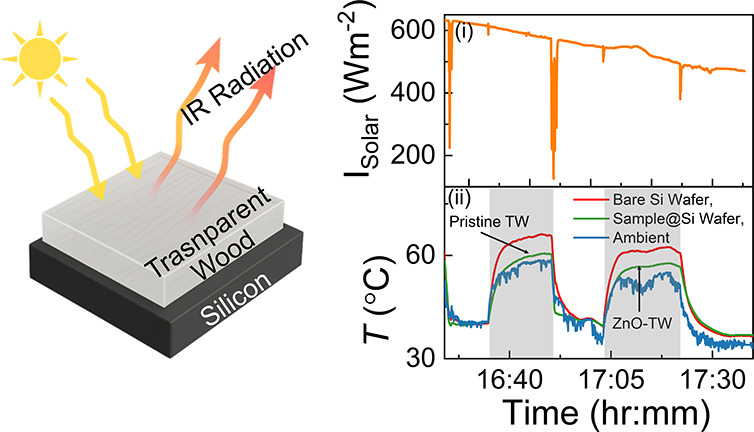

The demand for efficient thermal management solutions is growing rapidly due to rising global temperature and increasing urban heat.^1,2^ Conventional cooling methods, such as air conditioning, rely heavily on energy-intensive systems and contribute significantly to greenhouse gas emissions. This has motivated the development of passive cooling technologies that can reduce energy consumption, promoting environmental sustainability.^3–5^ Radiative cooling enables heat dissipation by allowing surfaces to emit thermal energy through the atmospheric transparency window (8–13 μm) into the cold deep space.^6–8^ Daytime radiative cooling therefore requires solar management and selective thermal emission (Figure S1) in the solar (0.28–2.5 μm) range, the surface should be either transparent (T≈1) or reflective (R→1) to avoid solar heating. In the atmospheric transparency window (8–13 μm), it should emit strongly (ε≈1) while remaining nonemissive elsewhere (ε≪1); by Kirchhoff’s law, absorptance equals emittance. Traditionally, subambient radiative cooling—where surface temperatures drop below ambient—has been achieved using materials with high solar reflectance and strong infrared emission.^9–11^ However, in applications where solar absorption must be maintained below a temperature limit, such as in silicon-based absorbers, the goal shifts from achieving subambient cooling to minimizing overheating while preserving optical performance.^12–14^ Conventional silica-based glass covers inherently provide limited radiative cooling due to their suboptimal infrared emissivity. To overcome this limitation, advanced solutions such as silica-based photonic crystals^15,16^ and microstructured silica layers,^17^ have been developed. Silica photonic crystals significantly enhance mid-infrared thermal emission with temperature reductions of up to 13 °C when integrated with silicon solar absorbers.^17^ Similarly, patterned silica microstructures achieved temperature reductions of about 3–5 °C while maintaining high transparency.^18^ While these approaches improve heat dissipation, challenges remain regarding their manufacturing complexity, durability challenges, potentially high cost, hindering their practicality and scalability.

Among organic passive cooling materials, cellulose stands out due to its intrinsic thermal emissivity, abundance, and biodegradability, making it an attractive candidate for eco-friendly cooling technologies.^19,20^ Delignified wood and cellulose films (both reflective and transparent), have successfully achieved cooling by scattering solar radiation and emitting strongly in the mid-infrared range, enabling temperature reductions of 5–9 °C under direct sunlight.^21–23^ While these materials are promising, their application in long-term cooling is hindered by mechanical fragility, scalability, and moisture sensitivity, limiting their practicality for long-term outdoor use such as coatings, which could be relevant for various architectural and energy-related surfaces. To overcome these challenges, transparent wood (TW) has emerged as a promising alternative.^24,25^ Impregnating chemically treated wood scaffolds with refractive index-matching polymers preserves the natural hierarchical structure, resulting in high mechanical strength, tunable optical properties, and improved environmental stability.^26,27^ Beyond its optical performance, TW offers a distinct advantage over glass, with a much lower thermal conductivity (0.19 W m^–1^ K^–1^), and 3 orders of magnitude greater mechanical toughness (3.03 MJ.m^–3^),^28^ than that of glass. These properties make it highly promising for energy-efficient applications, such as transparent radiative cooling and sustainable building designs. Recently, Hu et al. reported a self-adaptive radiative cooling system incorporating TW, as a mechanically robust and transparent substrate, topped with a Fabry–Perot multilayer coating based on tungsten-doped vanadium dioxide. In their design, radiative cooling was governed by the temperature-dependent emissivity of the coating, while TW served primarily as a passive support.^29^ In contrast, our focus is on utilizing TW itself as the functional cooling material—enhancing mid-infrared emission while maintaining solar transparency. TW biocomposites, typically a few hundred microns thick, uniquely combine optical transmittance with high mechanical toughness, making them well-suited for integration with heat-sensitive devices and solar-absorbing surfaces.^30,31^ When applied as thin-film coatings on silicon substrates, they lowered surface temperatures by ∼6–7 °C under direct sunlight, effectively mitigating overheating while maintaining optical functionality. Additionally, functionalization with nanoparticles such as ZnO into TW can further enhance mechanical robustness while maintaining sufficient optical clarity and cooling function for practical use. Our findings suggest that TW coatings could offer a practical and sustainable way to reduce the temperature of solar-absorbing surfaces in outdoor environments.

Figure 1a illustrates the preparation process of the TW-based radiative cooler, highlighting a three-step top-down fabrication approach. In the first step, native birch wood is bleached using a sodium citrate/H_2_O_2_–NaOH solution at 60 °C for 1–2 h to selectively remove chromophores while preserving hierarchical wood structure and retaining a significant portion of lignin. The resultant porous wood template preserves mechanical integrity for further functionalization. In the second step, bleached wood scaffold is functionalized with ZnO nanoparticles via vacuum infiltration of a ZnO nanosol. In the final step, the ZnO-functionalized scaffold (ZnO@bleached wood) is impregnated with a thiol–ene polymer precursor, followed by in situ UV polymerization. This polymerization proceeds via a step-growth mechanism, minimizing shrinkage strain during curing and promoting strong interfacial adhesion (scheme S1). Figure 1b presents the operational principle of the TW-based radiative cooler. The ZnO functionalized-TW (ZnO-TW) biocomposite is applied as a thin film on a silicon substrate, enabling passive radiative cooling by efficiently transmitting solar radiation and emitting thermal radiation within the 8–13 μm window. In Figure 1c, the left panel shows bleached wood template, which appears bright white due to the removal of chromophores and strong scattering of visible light caused by refractive index mismatches within its porous, fibrous structure. The right panel shows ZnO-TW biocomposite, which becomes optically transparent after in situ polymerization, enabled by refractive index matching, while retaining the natural hierarchical wood structure. The total transmission spectra in Figure 1d compare the optical performance of ZnO-TW biocomposite with 1 mm-thick glass. ZnO-TW biocomposite exhibits a transmittance of ∼84% at 550 nm, slightly lower than that of glass. This remarkable transparency in ZnO-TW biocomposite is achieved through precise refractive index matching between the thiol–ene polymer matrix (n ≈ 1.54) and the wood scaffold, minimizing interfacial scattering and ensuring efficient light transmission.^27^ The inset photograph further illustrates the clarity of ZnO-TW biocomposite and glass.

**Figure 1:**
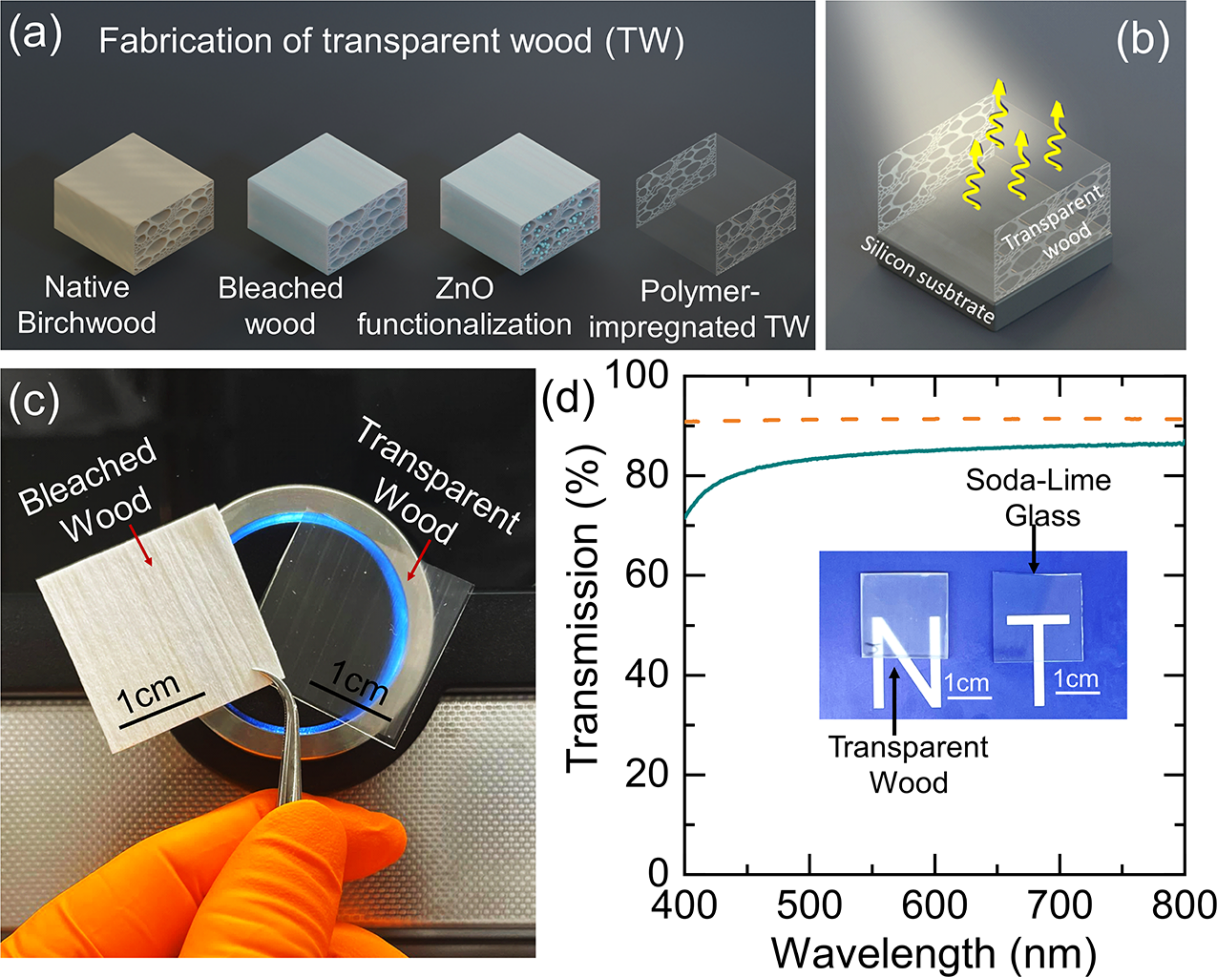
Schematic illustration of the fabrication process and characterization of a ZnO-TW biocomposite. (a) Schematic illustrating the step-by-step preparation of the biocomposite: bleaching of native wood, its ZnO functionalization, and final thiol–ene polymer precursor impregnation and its in situ UV polymerization. (b) Conceptual schematic of passive radiative cooling, showing ZnO-TW biocomposite coating on a silicon emitting thermal radiation in the atmospheric transparency window. (c) Digital photographs comparing bleached wood (opaque white) and the resulting ZnO-TW biocomposite (transparent). (d) Transmission spectra of ZnO-TW biocomposite (solid green line) and 1 mm-thick glass (orange dashed line), with ZnO-TW biocomposite achieving ∼84% transmittance at 550 nm. Inset: a digital image comparing the ZnO-TW biocomposite and glass.

The microstructure of wood governs the optical and mechanical properties of TW biocomposites. SEM was used to characterize the microscopic structure of all samples. Figure S2a shows the radial surface of bleached wood having cut wood fiber lumens and wood fiber surfaces, which are homogeneously decorated with spherical ZnO nanoparticles (diameter ∼20–40 nm, measured by ImageJ software) after ZnO functionalization (Figure S2b). The inset images in Figure S2a and S2b show the magnified wood fiber surface. In TW biocomposites, in situ thiol–ene polymerization successfully retained the wood structure and the wood fiber lumens are completely filled with the polymer matrix (Figure 2). Figures 2a and 2b display the cross sections of pristine TW (pristine TW) and ZnO-TW biocomposite structures, with insets providing a closer view of the polymer-filled cell walls and lumen spaces. Some debonding between the polymer and wood cell walls is visible in the SEM images, may have been caused during ultramicrotome sectioning of the TWs.^32^ To verify uniform polymer infiltration, S and Zn elemental mapping were performed on the ZnO-TW biocomposites (Figure 2c-e) using energy dispersive X-ray spectroscopy (EDS), which suggests the presence of S atoms in the wood fiber lumen and cell walls, confirming uniform infiltration of polymer precursor and their polymerization in the whole wood structure. Further, Zn mapping revealed that ZnO nanoparticles are homogeneously distributed within the wood structure, inside the wood cell wall. Thermogravimetric analysis (TGA) quantitatively supported this, showing ∼ 8% ZnO loading (Figure S3). This ZnO loading may enhance the mechanical properties of ZnO-TW biocomposite, partially block harmful UVA radiation, and selectively reflect shortwave to near-infrared (SWNIR ∼ 0.74–2.5 μm) wavelengths. These aspects are analyzed in detail in later sections.

**Figure 2:**
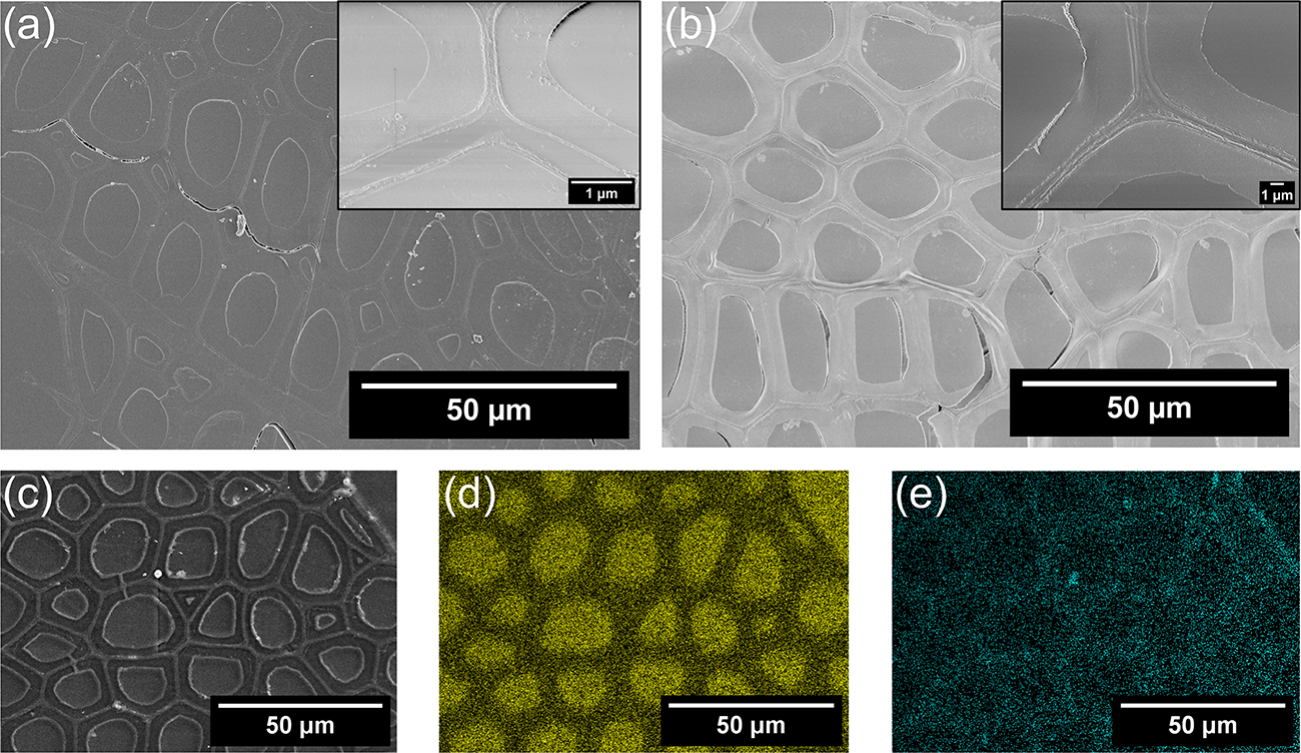
SEM analysis of TWs. SEM images of (a) pristine TW biocomposite and (b) ZnO-TW biocomposite with insets showing magnified cell walls and uniform thiol–ene polymer impregnation. EDS analysis of (c) ZnO-TW biocomposite, scanned site, (d) corresponding S mapping showing impregnation of thiol–ene polymer inside the cell walls and fiber lumens, and (e) Zn mapping showing the presence of ZnO NPs inside the wood fiber cell wall and lumen space.

For radiative cooling, it is essential to minimize absorption in the solar spectrum while maximizing emissivity in the mid-infrared region, particularly within the 8–13 μm atmospheric window. Since emissivity (ε) and absorptance are equivalent under thermal equilibrium (Kirchhoff’s law), we evaluated spectral absorptance by measuring total reflectance (*R*(λ)), and transmittance (*T*(λ)) using an integrating sphere (schematic shown in Figure 3a), which accounts for both specular and diffuse scattering. Absorptance was then computed as *A*(λ)=1–*R*(λ)–*T*(λ). The resulting spectra (Figure 3b) show the reflectance, transmittance, and absorptance spectra of pristine TW biocomposite (dashed lines) and ZnO-TW biocomposite samples (solid lines). The shaded AM1.5 solar spectrum and atmospheric IR transmission window are plotted in the background as guides to assess spectral selectivity and provide spectral context for interpreting the absorptance/emissivity profiles. Both samples exhibit absorptance below 5% throughout the 0.3–1.3 μm range, effectively minimizing solar energy absorption, and maintain high transmittance across the visible region (400–800 nm). At 550 nm, the ZnO-TW biocomposite (thickness ∼ 500 μm) achieves ∼ 84% transmittance, slightly lower than the ∼ 87% observed for pristine TW, due to the incorporation of ZnO nanoparticles. The higher refractive index of ZnO likely introduces additional scattering at the wood-polymer interfaces, together with possible optical defects such as interfacial gaps (Figure 2a,b), which may contribute to the observed reduction in transmittance and slightly increase in reflectance (∼14% for ZnO-TW vs ∼11% for pristine TW at 550 nm), while the difference in absorptance remains minimal (∼3%). These results indicate that ZnO functionalization preserves the material’s transparency with modest changes in scattering behavior.

**Figure 3:**
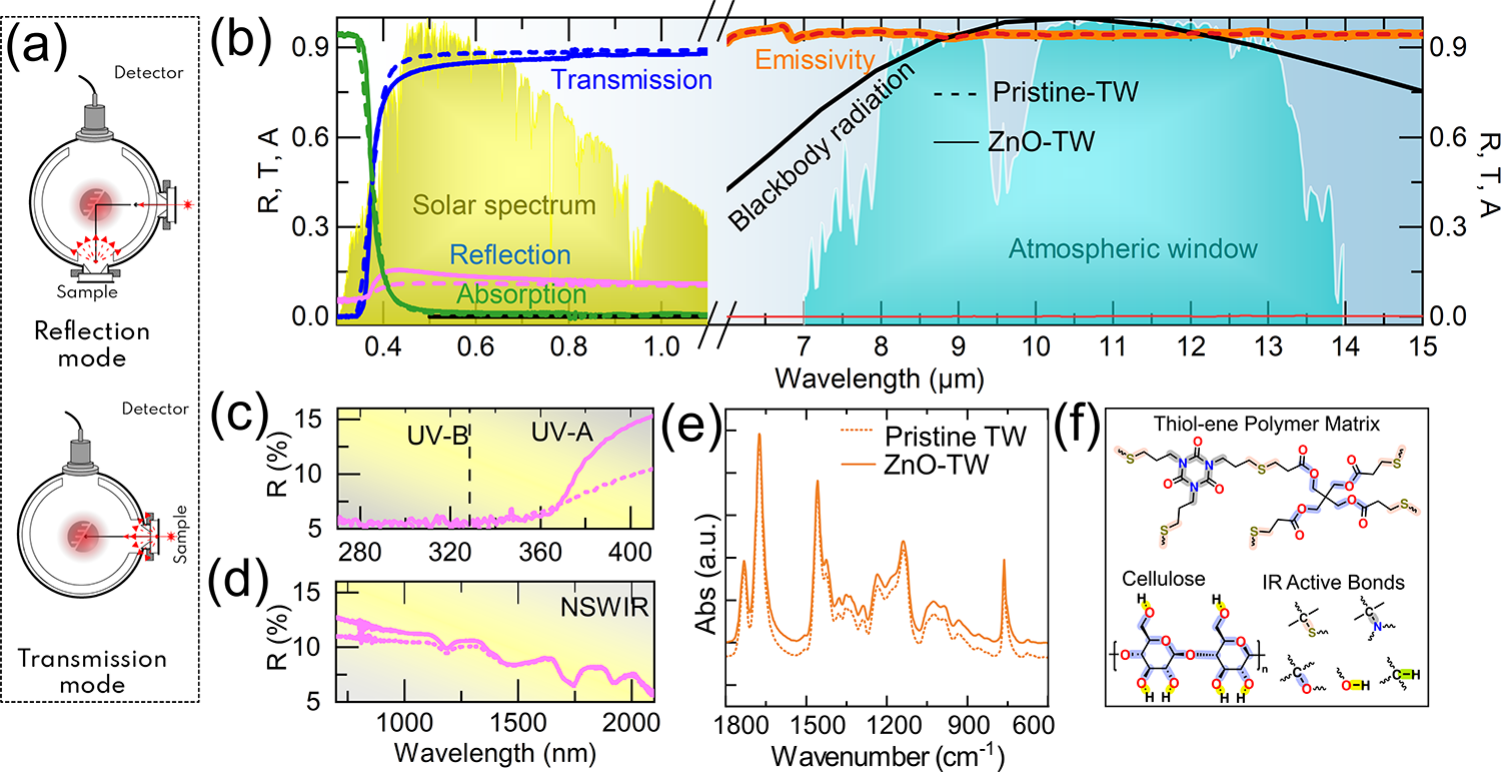
Spectral and emissive properties of the TW biocomposites, both pristine (dashed lines) and ZnO-TW (solid lines): (a) Schematic of the integrating sphere configuration used to measure total reflectance, transmittance, and absorptance for both pristine- and ZnO-TW biocomposite samples. (b) Spectral absorptance/emissivity of the biocomposites plotted alongside the AM1.5 solar spectrum (yellow) and the mid-IR atmospheric transparency window (violet-blue). Both samples exhibit low solar absorptance (<5%) from 0.3 to 1.3 μm and high mid-IR emissivity (∼95%) across the atmospheric window. (c) Zoomed-in reflectance in the UV range (280–400 nm), showing slightly higher reflectance for ZnO-TW in the UV-A region, suggesting additional UV-blocking functionality. (d) Reflectance in the near-infrared (900–2000 nm), where ZnO-TW shows modestly enhanced reflection, potentially reducing unwanted heat gain from NIR radiation. (e) FTIR spectra of TW biocomposites and their precursor templates, and (f) corresponding mid-IR active chemical bonds along with the molecular structure of the TW biocomposites’ constituents.

Figure 3b also presents the emissivity spectra of both pristine (dashed line) and ZnO-TW samples (solid line), showing high emissivity (∼95%) across the mid-IR atmospheric transparency with minimal spectral differences between the two, indicating that the addition of ZnO does not compromise the cooling potential. To further evaluate spectral selectivity, Figures 3c and 3d show zoomed-in reflectance profiles in the UV and near-infrared regions. Figure 3c reveals a slight increase in reflectance in the UV-A region for ZnO-TW, supporting its potential as a UV-blocking material—an added benefit of nanoparticle incorporation. Figure 3d shows enhanced reflectance in the near-infrared (NIR) range, which could further help in reducing unwanted heat absorption from nonvisible solar radiation. While modest in magnitude, these effects illustrate the multifunctional optical role of ZnO within the biocomposite. To understand the origin of the high mid-infrared emissivity observed in Figure 3b, we analyzed the molecular vibrational absorption features of the individual components using Fourier transform infrared (FTIR) spectroscopy. As shown in Figure 3e, the high emissivity observed in the TW biocomposites arises from the inherent infrared vibrational resonances through vibrational modes of cellulose, such as C–O stretching, centered around ∼1050 cm^–1^ (9.52 μm), which coincide with the atmospheric transparency window (8–13 μm). Additionally, the thiol–ene polymer matrix introduces S–H and C–S bonds (Figures 3d, 3e and S4). Together, these functional groups (Figure 3f) ensure broad (600–3500 cm^–1^ → 16.67–2.85 μm) and efficient thermal emission, making the biocomposite highly effective for radiative cooling applications.

To assess cooling performance of pristine and ZnO-TW biocomposite samples, we performed outdoor measurements under clear skies. The chamber’s inner walls were lined with reflective aluminum to limit parasitic solar heating, and a polyethylene (PE) film—transparent in the solar and mid-IR—suppressed convective exchange with the surroundings. Ambient temperature denotes the air inside the enclosure, capturing the local environment under reduced convection. We do not use this value to claim subambient cooling; it serves only as the baseline for comparison with a reference substrate measured simultaneously under identical conditions (Figure 4a). Pristine- and ZnO-TW biocomposites were placed on silicon wafers alongside an uncoated silicon refrence to ensure consistent substrate material and uniform environmental conditions. Shaded gray regions in the figure represent periods when a PE shutter covered the chamber to stabilize the environment and block direct solar exposure. The top panel of Figure 4b shows the measured solar irradiance (*I*_solar_), while the bottom panel presents the temperature profiles of Pristine- and ZnO-TW biocomposites, bare silicon reference, and ambient chamber temperature. The bare silicon consistently exhibited the highest temperatures, reaching just above 60 °C under peak irradiance, and remained significantly warmer than the ambient conditions (∼53–57 °C) throughout the measurement period. In contrast, both the pristine- and ZnO-TW biocomposites exhibited comparable temperature reductions relative to the bare reference, with peak differences reaching up to ∼6–7 °C depending on the biocomposite and during their respective measurement periods. Figure 4c shows ΔT profiles between the bare silicon and each coated sample, clearly illustrating the reduction in heat accumulation enabled by the coatings. This effect arises from their low visible-range absorption combined with high thermal emissivity in the 8–13 μm atmospheric window. We estimated net cooling power *P*_net_ (details in Section 1, Supporting Information) under varying ambient conditions and heat transfer coefficients *h*_*c*_ using the following eq 1:

1 $$P_{\mathrm{net}}=P_{\mathrm{rad}}-P_{\mathrm{atm}}-P_{\mathrm{nonrad}}-P_{\mathrm{solar}}$$

where *P*_rad_ is the thermal radiation power per area of the cooler, *P*_atm_ is the power per area absorbed by the cooler due to incident radiation from the atmosphere, *P*_nonrad_ accounts for power lost or gained due to conduction and convection, and *P*_solar_ corresponds to incident absorbed power per area from solar irradiation. Figure 4d shows the estimated net cooling power for both coated and bare silicon surfaces under different ambient temperatures and convective heat transfer coefficients based on calculations described in Supporting Information (Section 1). The coated sample consistently exhibits higher cooling power across all cases, explaining the temperature reduction observed in Figure 4c. As shown in Figures S5 and S6, this advantage arises from significantly higher radiative losses from the coated surface compared to the bare Si surface. This greatly compensates a minor increase in solar absorption (∼9 W m^–2^) upon adding the TW, thanks to the increase in thermal emissivity in the atmospheric window. We also assessed weather sensitivity by varying an effective LWIR atmospheric transmittance (clear/humid/overcast) and the convective coefficient; details in SI Section 1. As Figure S7a–c show that humidity/cloud cover and stronger convection shrink the cooling margin, but the coating retains a temperature advantage over bare Si.

**Figure 4:**
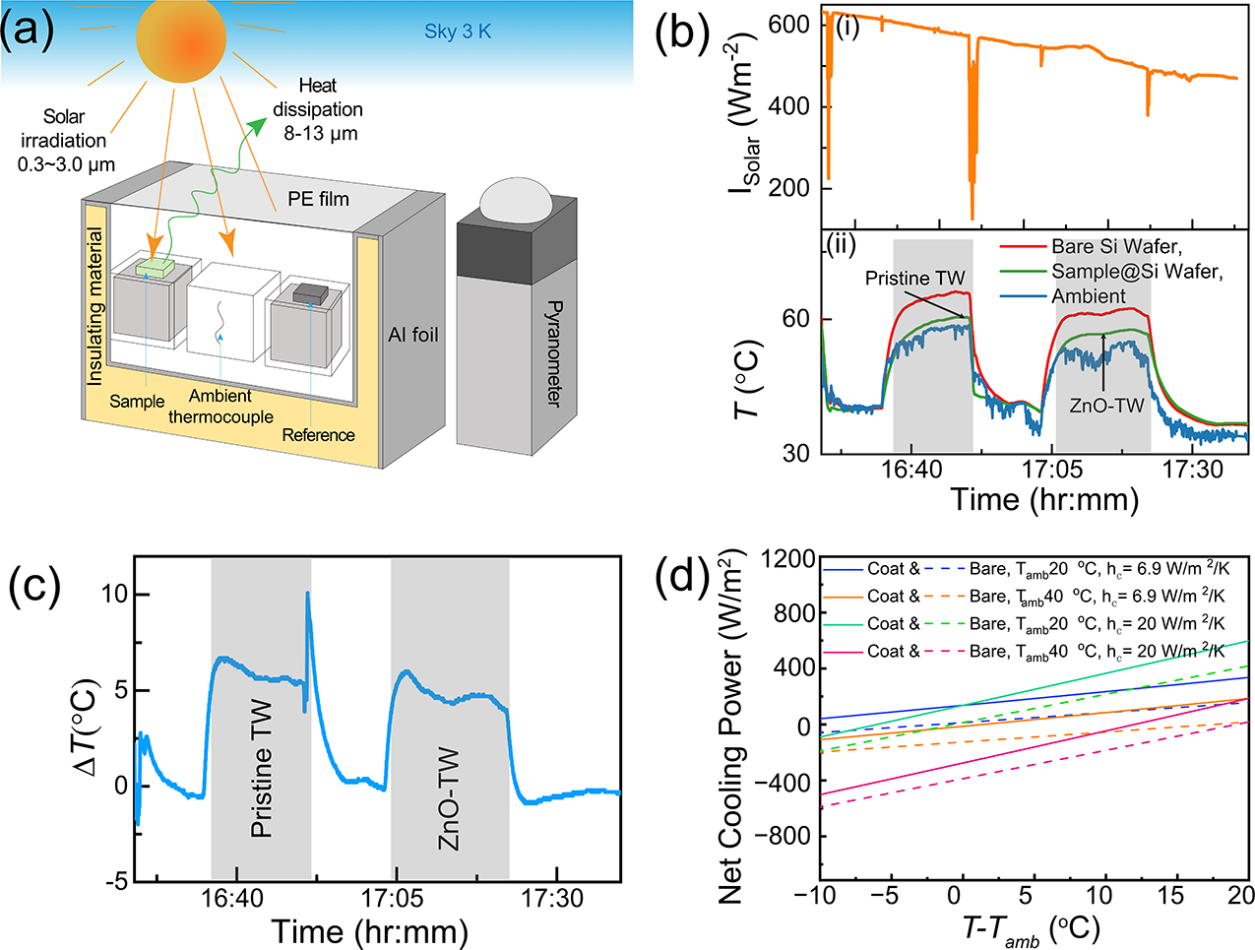
(a) Schematic of the measurement chamber used for evaluating radiative cooling performance, featuring a reflective aluminum foil lining and a PE film to minimize external environmental effects. (b) Measured solar irradiance (top panel), temperature profiles of the pristine- and ZnO-TW biocomposite-coated samples placed on silicon wafers, alongside the uncoated silicon reference and ambient temperature (bottom panel), (c) temperature differences (Δ*T*) between the coated samples and the uncoated reference. (d) Calculated net cooling power density for ZnO-TW biocomposite at different *T* – *T*_amb_.

This aligns with our observations and confirms that our coating effectively lowers the substrate temperature relative to an uncoated surface, even though it does not achieve subambient temperatures. Table S1 provides a comparative overview of our material’s radiative cooling performance alongside previously reported transparent cooling materials. Although direct comparisons are complicated by differences in solar irradiance, transparency, and thickness, our biobased wood composite demonstrates a promising combination of optical properties (high emissivity ∼ 0.95 and notable transparency of 84% at 550 nm wavelength), coupled with inherent advantages in mechanical robustness, moisture stability, and sustainability. These additional features, explored further in the next section, highlight the unique suitability of our composite as a transparent radiative cooling material.

To gauge durability, we assessed TW biocomposites’ mechanical properties via three-point flexural testing. The flexural stress–strain behavior of all samples is presented in Figures 5, and S8. ZnO-TW biocomposite achieves the highest flexural strength (158 ± 18 MPa) and modulus (13.6 ± 1.1 GPa), showcasing the reinforcing role of ZnO functionalization (Figure 5a). The pristine-TW biocomposite demonstrates notable mechanical properties, with a flexural strength of 130 ± 31 MPa and modulus of 12.8 ± 2.2 GPa, highlighting the contribution of the wood scaffold alone. The bleached wood and ZnO@bleached wood samples exhibit flexural strengths of 116 ± 3.1 MPa and 117 ± 12 MPa, respectively, with corresponding moduli of 13.1 ± 0.4 GPa and 15.4 ± 1.5 GPa (Figure S8). These values reflect the inherent strength of the wood scaffold, with only modest enhancement observed upon ZnO functionalization in the absence of polymer impregnation. In contrast, the neat thiol–ene polymer shows the lowest mechanical performance, with a flexural strength of 37.3 ± 7.6 MPa and modulus of 3.2 ± 0.3 GPa, underscoring the critical reinforcement provided by the wood template (Figure S8). ZnO-TW biocomposite resulted in a 21% increase in flexural strength, a 6% increase in modulus, and a ∼190% increment in toughness (work to fracture) over pristine-TW biocomposite, further demonstrating effectiveness of ZnO functionalization as a reinforcing agent. TW biocomposites offer high strength (130–158 MPa) over conventional soda-lime glass panels (40–90 MPa), and flexural strain of up to ∼ 1.5% at the break, resulting in higher toughness (work to fracture) (Figure 5b), 0.70, and ∼1.33 MJm^–3^ for pristine- and ZnO-TW biocomposites over the soda-lime glass (∼0.003 MJ m^–3^).^28,33^

**Figure 5:**
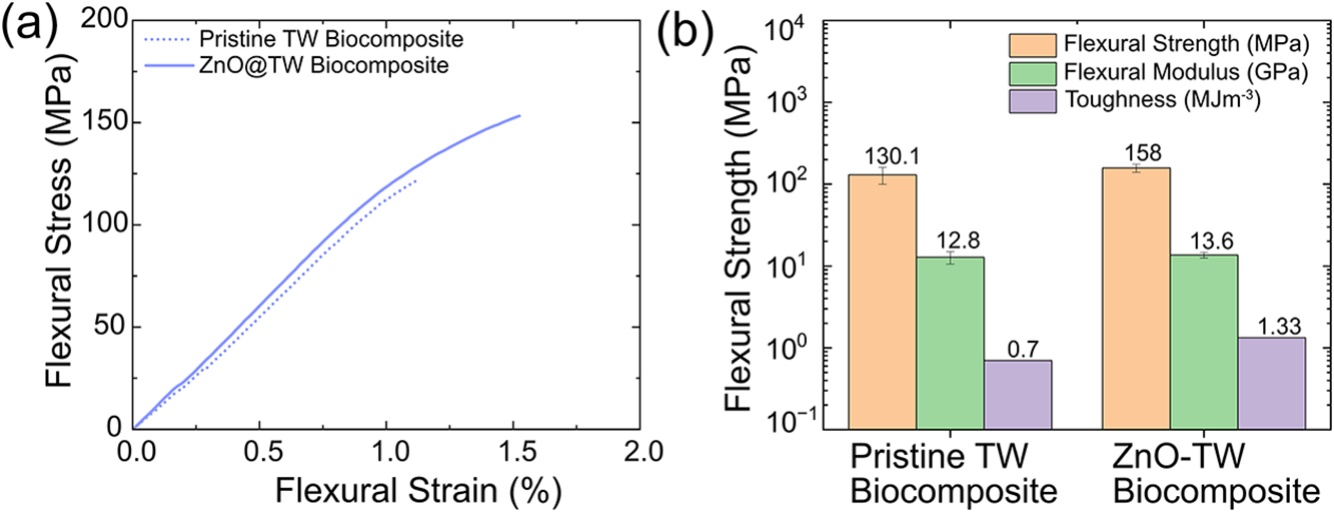
Mechanical performance of various wood scaffold samples and thiol–ene polymer matrix. (a) Flexural stress–strain curves for pristine- and ZnO-TW biocomposites. (b) Comparison of flexural strength and modulus, showing ZnO-TW biocomposite’s enhanced properties due to ZnO functionalization and the wood scaffold’s reinforcement.

These results underline the synergistic impact of the wood template and ZnO functionalization in producing biocomposites with good mechanical properties, making them suitable for passive radiative cooling applications, where structural integrity (in terms of both strength and durability) is also needed such as transparent roofs, solar panel coverings, etc. Table S1 also compares mechanical properties of TWs with other transparent cooling systems. While differences in testing conditions and sample thicknesses may affect direct comparisons, the combination of high visible transparency (∼84%) and strong thermal emissivity (∼0.95), together with mechanical resilience, scalable fabrication, and environmental stability, highlights its potential for sustainable thermal management applications. In addition, cross-linked thiol–ene polymers are known for their UV and moisture stability,^34^ and previous reports on epoxy-impregnated TW systems as well as epoxy surface coatings on wood have reported for their long-term outdoor durability.^35,36^

This study presents a passive radiative cooling strategy using TW biocomposites functionalized with ZnO and impregnated with a thiol–ene polymer. The resulting material combines high visible transmittance (∼84%), strong mid-infrared emissivity (∼0.95), and enhanced mechanical strength. When applied to silicon substrates, the TW coating reduced surface temperatures by up to 6–7 °C under direct sunlight in outdoor tests. This cooling effect is attributed to low solar absorptance and efficient thermal emission within the 8–13 μm atmospheric window. In contrast to conventional cooling strategies that rely on multilayer designs or nanofabrication, our approach utilizes naturally derived materials and straightforward processing. The ZnO-TW biocomposite demonstrates excellent mechanical performance, with a flexural strength of 158 ± 18.1 MPa, a modulus of 13.6 ± 1.1 GPa, and toughness (work to fracture) (∼0.70 to 1.33 MJm^–3^) exceeding strength and toughness in conventional monolithic materials like soda-lime glass. This level of strength, combined with high optical performance and outdoor durability, makes TW a promising candidate that is scalable and eco-friendly for passive thermal management in solar-exposed environments such as rooftops, facades, and photovoltaic modules.

## Supplementary Material



## Notes

Materials, procedures for making TW composites, methods of characterization, reaction schematic of polymerization, SEM and TGA of wood templates, FTIR and mechanical properties of thiol–ene polymer matrix and wood templates, radiative exchange and solar gain for TW composite coated and bare Si, optical properties of bare Si, comparison table of transparent PDRCs, effect of humidity/cloud cover and stronger convection on radiative cooling and cooling power density estimation calculation (PDF)
