# Supplementary material for: Transparent Wood for Passive Radiative Cooling of Solar Absorbers
Source: Nano Lett. 2025 Sep 11;25(38):14025–31. doi: 10.1021/acs.nanolett.5c02994 (PMC12464995; doi:10.1021/acs.nanolett.5c02994)
Supplement: Supplementary file 1 [file nl5c02994_si_001.pdf]

Supporting info for

Transparent Wood for Passive Radiative Cooling of Solar Absorbers

Farsa Ram[1,2], Martin Höglund[1], Migna Liao[3], Tomas Hallberg[4], Magnus Jonsson[3], Lars A. Berglund[1]*, Ravi Shanker[1]*

[1]Wallenberg Wood Science Center, Department of Fiber and Polymer Technology, KTH Royal Institute of Technology, Teknikringen 56, 100 44 Stockholm, Sweden
E-mail: blund@kth.se, shanker@kth.se
[2]Interdisciplinary Centre for Energy Research, Indian Institute of Science, Bengaluru-560012, India

[3]Laboratory of Organic Electronics, Department of Science and Technology, Linköping University, SE-601 74 Norrköping, Sweden

[4]FOI-Swedish Defense Research Agency, Department of Electro-Optical Systems, 583 30 Linköping, Sweden

Keywords: Passive radiative cooling, thermal radiation, thiol-ene, transparent wood, atmospheric window, cellulose, zinc oxide

This file includes:

Pages S1-S16

Scheme S1

Figures S1-S8

Table S1

Section §1

Experimental Section

Materials: Native wood (*Betula pendula*) veneers with a thickness of ~ 0.5 mm were purchased from Calexico Wood AB, Sweden. Sodium hydroxide, absolute ethanol, and acetone were purchased from VWR, Sweden. Sodium citrate, hydrogen peroxide (30%), zinc acetate dihydrate, zinc nitrate hexahydrate, hexamethylenetetramine (HMTA), pentaerythritol tetrakis(3-mercaptopropionate) (PETMP, tetrafunctional thiol monomer), 1,3,5-triallyl-1,3,5-triazine-2,4,6(1H,3H,5H)-trione (TATATO, trifunctional ene monomer), 1-hydroxycyclohexyl phenyl ketone (UV sensitive radical initiator), were supplied from Sigma-Aldrich, Sweden. All chemicals were used as received without any further purification.

Preparation of Transparent Wood Biocomposites and their Templates

Preparation of bleached wood: Native wood templates (5 x 6 cm^2) were cut from a large wood sheet and subjected to bleaching. Bleaching treatment allows lignin chromophore modification while preserving the structural integrity of the wood scaffold. The bleaching was performed by submerging the wood templates in a bleaching solution (hydrogen peroxide (6 wt%), sodium hydroxide (1 wt%), and sodium citrate (1 wt%) in deionized water) at 60 °C for 2h.[1] The samples were washed with DI water, and sequentially, the solvent was exchanged by varying ethanol concentration from 25% to 100%. In the last step, ethanol was exchanged with acetone. The samples were stored in acetone for further use.

ZnO-functionalized wood or ZnO@bleached wood was prepared by slightly modifying a method reported, previously.[2] Briefly, zinc acetate and sodium hydroxide were dissolved in ethanol to prepare 100 mM solutions. ZnO nanoparticles sol was prepared by dropwise addition of sodium hydroxide solution into the zinc acetate solution at 60-70 °C until the solution started to turn cloudy, which indicates the formation of colloidal ZnO nanoparticles in the solution. The solution was then immediately cooled to room temperature, and the bleached wood

samples were transferred into a portion of the ZnO NPs sol and subjected to vacuum infiltration for 30 min, where the ZnO NPs infiltered into the wood scaffold. The samples were dried in the oven at 110 °C for 3 min. The vacuum infiltration and drying steps were repeated 3 times, and finally, the ZnO NPs functionalized samples (ZnO-bleached wood) were stored back into acetone. These samples were kept in acetone for at least 72 hours before transparent wood preparation to ensure sufficient wetting.

Transparent wood preparation: Transparent wood was prepared by following previously reported method.[3] The bleached wood and ZnO@bleached wood templates (in their wet state) were infiltered with a stoichiometric mixture of thiol and ene monomers (PETMP and TATATO) along with a UV-initiator1-hydroxycyclohexyl phenyl ketone (0.5 wt %). The templates were soaked in the polymer precursor solution overnight, followed by vacuum filtration for 2 h to ensure complete impregnation of polymer precursor into the hierarchical wood structure. Finally, the samples were sandwiched between glass slides and cured for 2 min on each side of the sample by shining a 365 nm UV irradiation using a 9 W lamp. Vacuum infiltration and in situ UV curing formed a robust thiol–ene polymer network within the scaffold. The samples were peeled off from the glass plate and termed as transparent wood (TW) biocomposites, pristine-TW biocomposite (when only bleached wood template was used), and ZnO-TW biocomposite (when ZnO functionalized bleached wood template was used).

Material Characterization

The morphologies of the samples were observed with a Field-Emission Scanning Electron Microscope (FE-SEM, Hitachi S-4800, Japan) operating at an acceleration voltage of 3 kV. Cross-sections of transparent wood biocomposites were carried out using a Leica Ultracut UCT with a 45° diamond knife (DiATOME, Switzerland). Pt/Pd coating was sputtered on the

samples for 40 seconds before characterization. The energy dispersive X-ray spectroscopy (EDS) equipped on the FE-SEM (Oxford Instruments, X-MAX N 80, UK) was used for elemental mapping. Thermogravimetric analysis (TGA, Mettler Toledo- TGA/DSC 1, Switzerland) was performed to measure the ZnO loading at a heating rate of 10 K/min from room temperature to 800 °C under O_2 flow of 50 mL/min. The functional groups were characterized at room temperature by FTIR using a PerkinElmer spectrum 100 FT-IR equipped with an MKII Golden Gate single-reflection accessory unit with a diamond ATR crystal (Graseby Specac Ltd., UK). The 3-point bending test was performed to measure the flexural strength of the samples. The samples were cut into 5 cm x 6 cm, and the test was carried out using a universal testing machine (Instron 5944, USA) using a 500 N load cell at a 10 % min^{-1} strain rate. The active area between the 3 points was 30 mm, and 5-6 specimens were tested for each kind of sample.

Spectral measurements – The optical properties of the samples, including reflectance, transmittance, and absorptance, were measured across a broad wavelength range using two spectrometers. A Cary 5000 spectrophotometer was used to cover the ultraviolet to near-infrared range (250–2500 nm), while a Bruker Vertex 70 Fourier Transform Infrared (FTIR) spectrometer measured in the mid- and far-infrared region (2–33 µm). Both instruments were equipped with integrating spheres to capture directional hemispherical reflectance (DHR) and transmittance (DHT) under near-normal incidence. For the Cary instrument, a Labsphere DRA-2500 integrating sphere was employed, while the FTIR system used a Labsphere A562 sphere. The detectors varied depending on the spectral range: a photomultiplier tube (PMT) for UV–VIS, a cooled PbS detector for NIR, and a deuterated triglycine sulfate (DTGS) detector for the infrared region. Reflectance measurements relied on calibrated standards, including Spectralon® for UV-VIS and Infragold® for infrared, ensuring accuracy across both instruments. Transmittance and reflectance data were combined to calculate the absorptance of

the samples, adhering to the conservation of energy principle. Emissivity, an important parameter for thermal radiation, was derived from absorptance. According to Kirchhoff's law, emissivity at a given wavelength is directly equal to the absorptance, which indicates how efficiently the material radiates heat in the infrared spectrum. For a perfect black body, emissivity would be 1, meaning no light is reflected or transmitted.

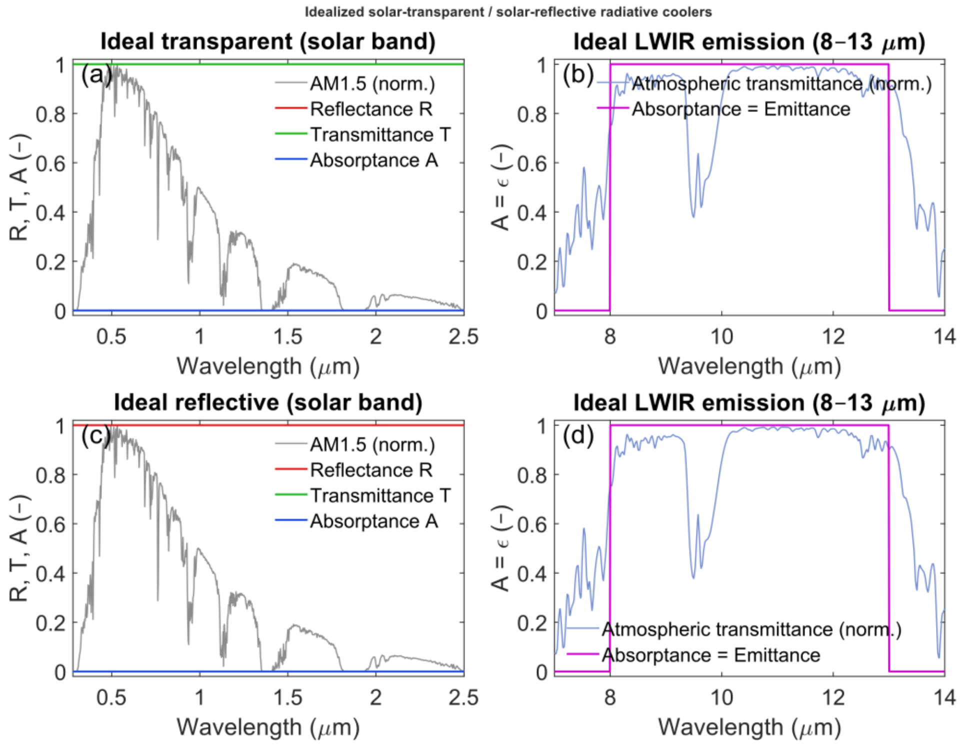

Figure S1: Ideal spectral requirements for passive daytime radiative cooling. (a) Solar-transparent: across the solar band (0.28–2.5 μm) the coating is ideally lossless and non-scattering (T→1, R≈A≈0), shown together with the normalized AM1.5 spectrum. (b) LWIR emission: absorptance (≈ emittance, by Kirchhoff's law) is unity only within the atmospheric window (8–13 μm) and ≈ 0 outside; the normalized atmospheric transmittance is overlaid to indicate the sky window. (c) Solar-reflective/scattering type: in the solar band the coating reflects sunlight (R→1, T≈A≈0), again shown with the normalized AM1.5 spectrum. (d) LWIR emission (shared requirement): same ideal top-hat emittance profile as in (b), matching the 8–13 μm window.

Thiol-Ene Polymerization Scheme

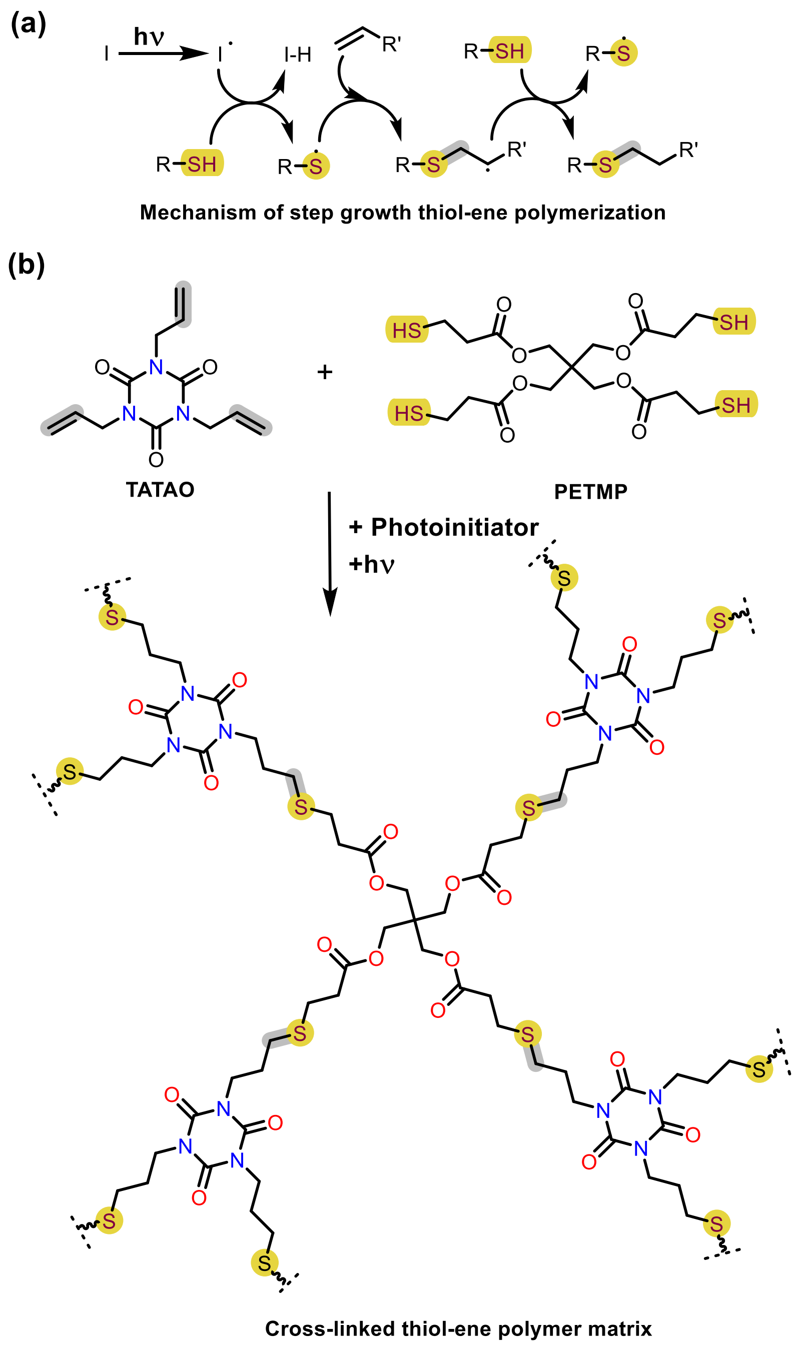

Scheme S1: (a) Mechanism of thiol-ene polymerization. In the initiation step, the initiator breaks in the presence of UV light and forms a radical initiator. In the propagation step, the initiator radical reacts with thiol and subtracts the hydrogen, resulting in the formation of the thiyl radical, which will react with ene monomer. The newly formed alkyl radical further reacts with thiol to generate thiyl radical. These steps go on until all the thiol and ene monomers are consumed. (b) Thiol-ene polymerization and resultant polymer matrix.

SEM images of wood templates

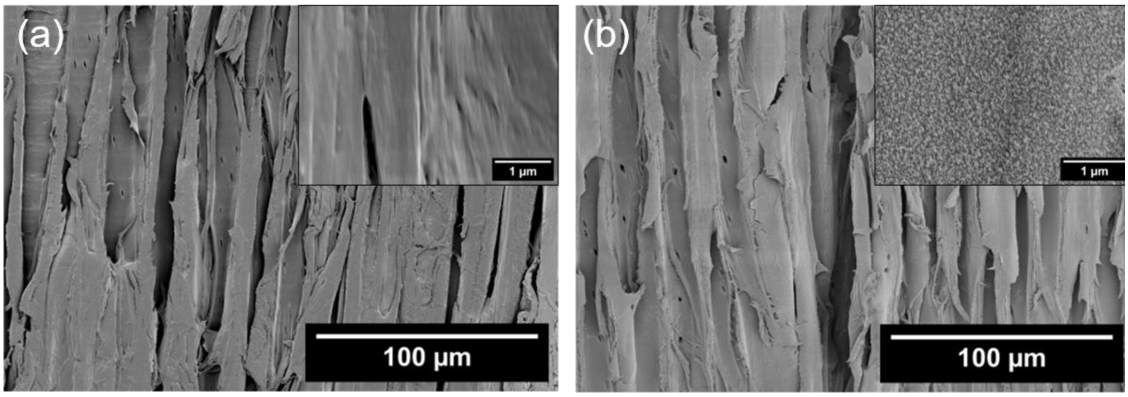

Figure S2: SEM images of (a) bleached wood template and (b) ZnO@bleached wood. The SEMs are of radial surface of wood substrate and insets show a zoomed-in portion of the respective templates.

The thermogravimetric analysis (TGA)

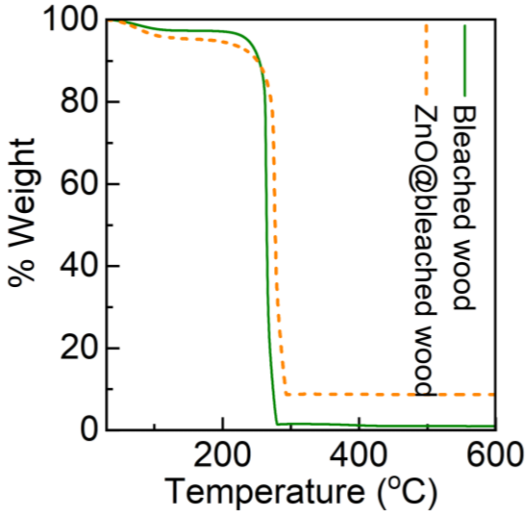

Figure S3. TGA curves comparing Bleached wood and ZnO@bleached wood, showing ~8% ZnO loading.

FTIR Analysis

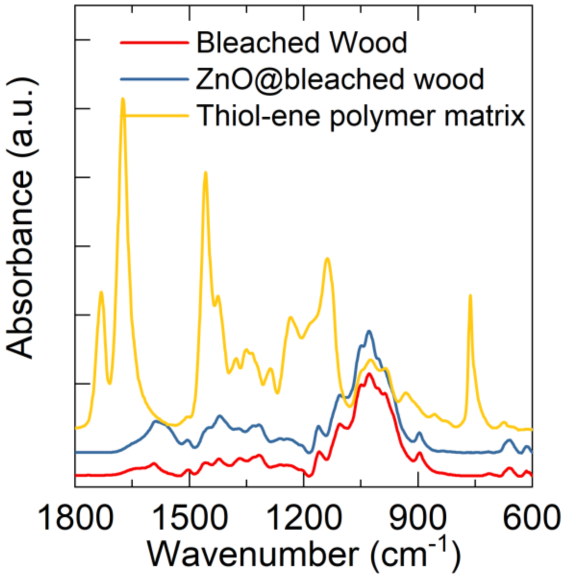

Figure S4: FTIR spectra of bleached wood, ZnO@bleached wood, and thiol-ene polymer matrix.

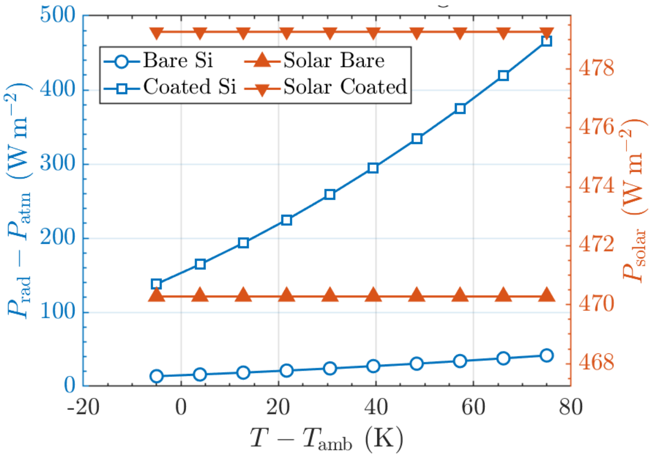

Figure S5: Breakdown of radiative exchange and solar gain for TW coated and bare silicon. The coated surface shows significantly enhanced radiative exchange at elevated temperatures, while the increase in solar gain is modest (~9 W/m²), resulting in a higher net cooling potential.

Radiative exchange and solar gain

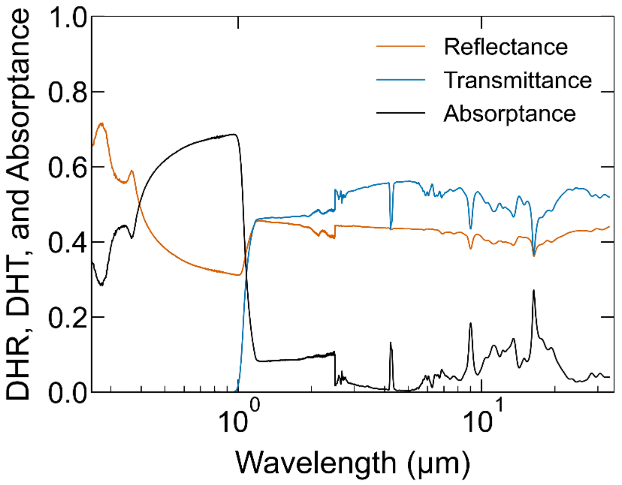

Figure S6: Experimental absorbance, reflectance, transmittance, and emissivity spectra of Si.

Weather and convection sensitivity

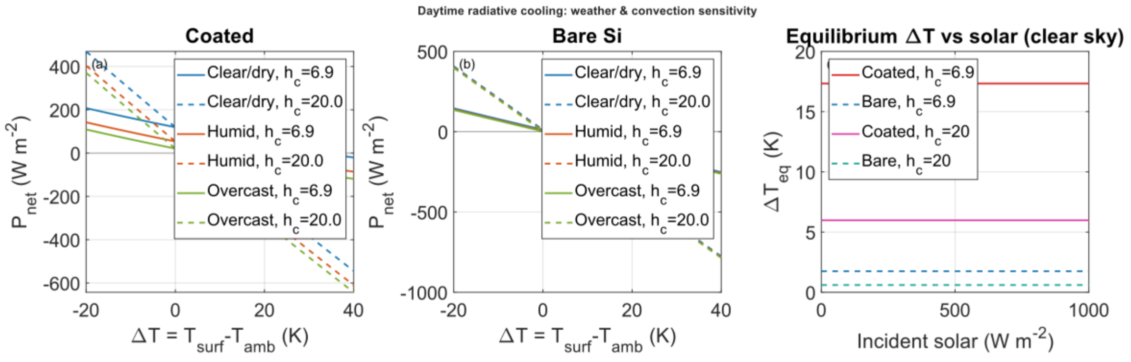

Figure S7: Weather and convection effects. (a) Coated: net cooling power vs. temperature under clear, humid, and overcast skies, as addressed by varying the atmospheric transparency (see details in Section §1). Solid lines = low convection; dashed lines = high convection. (b) Bare Si: same trends, but lower curves. (c) Equilibrium temperature vs. incident solar (clear sky).

Mechanical Properties

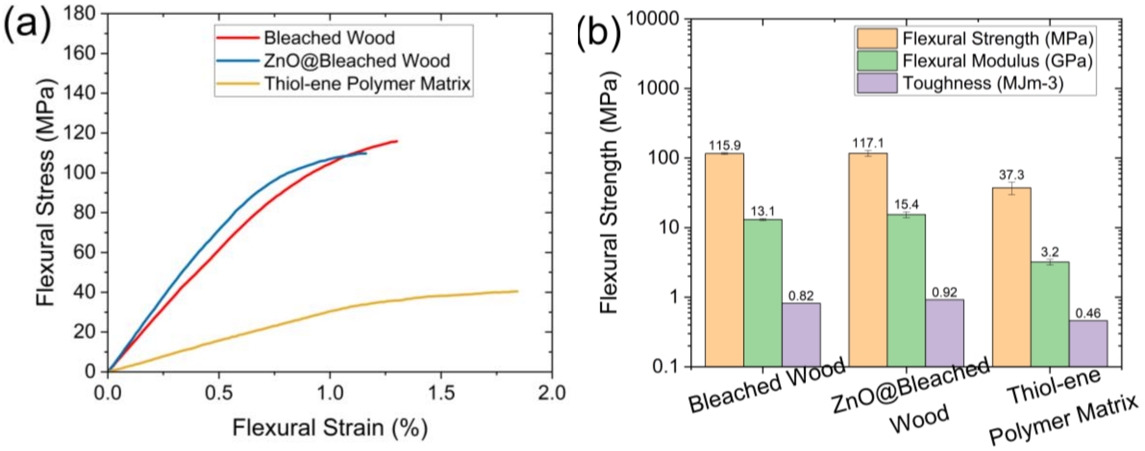

Figure S8: (a) Flexural stress–strain curve and (b) toughness for bleached wood, ZnO@bleached wood and thiol-ene polymer matrix.

Table S1: Comparison of transparent PDRCs, and their various properties

	Emissivity	Temperature Drop (°C)	Thickness (µm)	Transparency (%)	Elastic Modulus (GPa)	Toughness (MJm^{-3})	Cooling Power (W/m^2)	Ref
**Grating SiO_2 Structure (Si wafer)**	0.9	3.6	500	90	175	/	/	4
**Silk Fibroin (Solar Panel)**	0.88	5.1	60	91	1	/	77.6	5
**SiO_2 aerogel microparticle /PDMS (solar Cell)**	0.98	7.7	150	91	0.00261	/	113	6
**nanocellulsoe film (Si Wafer)**	0.85	5	65	90	46	/	/	7
**PMMA-SiO_2/polyimide**	0.95	8.3	50	85	2.51	/	109	8
**Pristine TWs (Si Wafer)**	0.95	6	500	87	12.8	0.70		**This Work**
**ZnO-TW (Si Wafer)**	0.95	7	500	84	13.6	1.33	/	**This Work**

Section §1

Cooling power density estimation

The net cooling power density, P_{net} was calculated following the methodology outlined previously by (Zaman et al. 2019),[9] and is expressed as:

$$P_{\text{net}} = P_{\text{rad}} - P_{\text{atm}} - P_{\text{nonrad}} - P_{\text{solar}} \qquad \text{Eq 1}$$

where P_{rad} is the thermal radiation power per area of the cooler, P_{atm} is the power per area absorbed by the cooler due to incident radiation from the atmosphere, P_{nonrad} accounts for power lost or gained due to conduction and convection, and P_{solar} corresponds to incident absorbed power per area from solar irradiation.

P_{rad} is given by:

$$P_{\text{rad}} = \int_{\Omega} \cos(\theta) \int_0^{\infty} I_{BB}(\lambda, T)\varepsilon(\lambda, \theta)\mathrm{d}\lambda\,\mathrm{d}\Omega \qquad \text{Eq 2}$$

where $I_{BB}(\lambda, T)$ is the thermal emission spectrum of a perfect black body at temperature T, Ω is the solid angle of a hemisphere and $\varepsilon(\lambda, \theta)$ is the emissivity (absorption) of the cooler, which we assume to be angle-independent and equal to the absorption of the cooling coating in our calculations. P_{rad} can then be calculated via the simplified equation:

$$P_{\text{rad}}(T) = \pi \int_0^{\infty} I_{BB}(\lambda, T)\varepsilon(\lambda)\mathrm{d}\lambda \qquad \text{Eq 3}$$

where we used $\int_{\Omega} \cos(\theta)\,\mathrm{d}\Omega = 2\pi \int_{\theta=0}^{\pi/2} \sin(\theta)\cos(\theta)\,\mathrm{d}\theta = \pi$ for integration over the hemisphere.

Similarly, P_{atm} can be obtained by

$$P_{\text{atm}}(T_{\text{amb}}) = \int_{\Omega} \cos(\theta) \int_0^{\infty} I_{BB}(\lambda, T_{\text{amb}})\varepsilon(\lambda, \theta)\varepsilon_{\text{atm}}(\lambda, \theta)\mathrm{d}\lambda\,\mathrm{d}\Omega$$

Eq 4

where T_{amb} is the ambient temperature and $\varepsilon_{\text{atm}}(\lambda, \theta)$ is the emissivity of the atmosphere, given by:

$$\varepsilon_{\text{atm}}(\lambda, \theta) = 1 - t_{\text{atm}}(\lambda)^{1/\cos(\theta)} \qquad \text{Eq. 5}$$

where $t_{\text{atm}}(\lambda)$ is the atmospheric transmittance at zenith ($\theta = 0$). We then get:

$$P_{\text{atm}}(T_{\text{amb}}) = 2\pi \int_0^{\pi/2} \sin(\theta)\cos(\theta) \int_0^{\infty} I_{BB}(\lambda, T_{\text{amb}})\varepsilon(\lambda, \theta)\,\varepsilon_{\text{atm}}(\lambda, \theta)\mathrm{d}\lambda\,\mathrm{d}\theta.$$

Eq. 6

In our calculations, we calculated P_{rad} and P_{atm} using the same wavelength range (7µm - 13.9µm) and using the atmospheric transmittance data from Atmospheric Transmission, Gemini Observatory, 2005 for the latter.[10]

P_{nonrad} can be estimated by:

$$P_{\text{nonrad}} = h_c(T_{\text{amb}} - T) = -h_c\Delta T \qquad \text{Eq. 7}$$

where h_c is the nonradiative heat transfer coefficient, which we set to 6.9 W/(m^{2}K) or 20 W/(m^{2}K) in our calculations.

Finally, P_{solar} is given by:

$$P_{\text{solar}} = \int_0^{\infty} I_{\text{solar}}(\lambda)\alpha(\lambda)\text{d}\lambda \quad \text{Eq. 8}$$

where $\alpha(\lambda)$ is the absorptance of the whole device and $I_{\text{solar}}(\lambda)$ is the effective solar irradiance on the cooler, which we estimate as the AM1.5 solar spectrum reduced to 0.8 sun based on measurements using a solar cell reference meter (Newport, model number 91150V) positioned next to the measurement system and with same angle to the sun as the coolers. P_{solar} was calculated over the wavelength range 280nm to 13.9μm. We accounted for solar absorption not only by the TW cooling material but also by the underlying silicon substrate using:

$$\alpha(\lambda) = A_{\text{coating}}(\lambda) + T_{\text{coating}}(\lambda) \cdot A_{\text{Si}}(\lambda) + T_{\text{coating}}(\lambda) \cdot R_{\text{Si}}(\lambda) \cdot A_{\text{coating}}(\lambda) \quad \text{Eq. 9}$$

where $A_{\text{coating/Si}}$, $T_{\text{coating/Si}}$, and $R_{\text{coating/Si}}$ correspond to measured absorptance, transmittance and reflectance, respectively, for the coatings or the underlying silicon substrate.

Weather emulation

We modeled weather sensitivity heat-balance model $P_{\text{net}}(\Delta T) = (P_{rad} - T_{\text{atm}}) - h_c\Delta T - P_{solar}$. Sky clarity was emulated by scaling the clear-sky atmospheric transmittance $t_{clear}(\lambda)$ in the 7-14 μm band as $t_{atm,eff}(\lambda) = clip[f t_{clear}(\lambda), 0,1]$, which gives the angle dependent atmospheric emissivity $\varepsilon_{atm}(\lambda, \theta) = 1 - t_{eff}(\lambda)^{\frac{1}{\mu}}$ with $\mu = cos\theta$. We take $f = 1.0$ (clear/dry), $f = 0.6$ (humid), and $f = 0.3$ (overcast). Intuitively: lower f → larger ε_{atm} and P_{atm}→ smaller (P_{rad} −P_{atm}) → warmer equilibrium (i.e., higher ΔT_{eq}), while larger h_c steepens the $-h_c\Delta T$ term and likewise pushes the operating point towards ambient. The absorbed solar term is $P_{\text{solar}} = \int s\, I_{AM1.5}(\lambda)\, \alpha(\lambda)\text{d}\lambda$, with s = 1 for clear/humid and s =0.4 for overcast.

References:

1. Wu, Yan, Jiamin Wu, Feng Yang, Caiyun Tang, and Qiongtao Huang. "Effect of H2O2 bleaching treatment on the properties of finished transparent wood." *Polymers* 11, no. 5 (2019): 776.

2. Ram, Farsa, Jonas Garemark, Yuanyuan Li, and Lars Berglund. "Scalable, efficient piezoelectric wood nanogenerators enabled by wood/ZnO nanocomposites." Composites Part A: Applied Science and Manufacturing 160 (2022): 107057.

3. Höglund, Martin, Mats Johansson, Ilya Sychugov, and Lars A. Berglund. "Transparent wood biocomposites by fast UV-curing for reduced light-scattering through wood/thiol–ene interface design." ACS Applied Materials & Interfaces 12, no. 41 (2020): 46914-46922.

4. Zhao B, et al. Radiative cooling of solar cells with micro-grating photonic cooler. Renewable Energy 191, 662-668 (2022).

5. Chen Y-H, et al. Eco-friendly transparent silk fibroin radiative cooling film for thermal management of optoelectronics. Advanced Functional Materials 33, 2301924 (2023).

6. Lee KW, et al. Visibly clear radiative cooling metamaterials for enhanced thermal management in solar cells and windows. Advanced Functional Materials 32, 2105882 (2022).

7. Gamage S, et al. Reflective and transparent cellulose-based passive radiative coolers. Cellulose 28, 9383-9393 (2021).

8. Lee, Kang Won, et al. Transparent radiative cooling cover window for flexible and foldable electronic displays. Nature Communications 15, 4443 (2024).

9. Zaman. M. Asif (2019) Photonic radiative cooler optimization using Taguchi’s method. International Journal of Thermal Science 144:21-26.

10. Atmospheric Transmission Data, Gemini Observatory, (2005) http://www.gemini.edu/sciops/instruments/mid-ir-resources/spectroscopic-calibrations/atmospherictransmission-data.
